# A narrative review of neuroprotective effects of remifentanil: from basic research to clinical practice

**DOI:** 10.3389/fneur.2025.1707972

**Published:** 2025-12-10

**Authors:** Xun Jiang, Meirong Wu, Chenghui Yan, Jia Liu

**Affiliations:** 1 Department of ICU (Intensive Care Unit), Qingbaijiang District People's Hospital of Chengdu City, Chengdu, Sichuan, China; 2 Department of Emergency, Qilu Hospital of Shandong University, Jinan, Shandong, China

**Keywords:** remifentanil, neuroprotection, apoptosis, neuroinflammation, ischemia–reperfusion injury

## Abstract

Opioid receptor agonists constitute a class of pharmaceuticals that are extensively employed in anesthesia and analgesia. Remifentanil (RF), a novel synthetic opioid receptor agonist, is characterized by rapid onset of action and a short half-life, attributed to its unique pharmacokinetic profile. Clinically, RF is often used for total intravenous anesthesia. In recent years, RF has garnered more attention due to its potential organ-protective effects. This review aims to consolidate the existing evidence regarding the neuroprotective properties of RF, encompassing various levels ranging from molecular mechanisms to clinical applications. Experimental studies demonstrate that RF exerts protective effects in brain injury models, with its mechanisms being associated with the attenuation of apoptosis, oxidative stress, and neuroinflammation. In clinical practice, RF serves as a safe and effective analgesic regimen for patients with traumatic brain injury (TBI), those undergoing neurosurgical procedures, and individuals requiring endotracheal intubation; furthermore, it confers certain benefits to the recovery of patients' neurological function. Compared with fentanyl, RF is capable of reducing the incidence of delirium and postoperative cognitive dysfunction (POCD) in patients. Moreover, remifentanil-induced hyperalgesia (RIH) during clinical administration is also discussed. In conclusion, RF is an anesthetic agent with significant neuroprotective potential. Future research should focus on elucidating its precise mechanism of action, optimizing clinical administration regimens, and exploring strategies in RIH management.

## 1 Introduction

Opioids are extensively utilized in clinical practice and exert their pharmacological effects through the activation of opioid receptors (OPRs), which are predominantly coupled with three major subtypes, namely, μ, κ, and δ (1). OPRs exhibit a broad distribution pattern across the cardiovascular system, central nervous system (CNS), and peripheral nerves. They are expressed in organs beyond the cardiovascular system, such as the gastrointestinal tract and kidneys (2). In the clinic, opioids are not only widely employed for the induction and maintenance of general anesthesia but also serve as pivotal agents for analgesic therapy in the management of acute pain and cancer-related pain. However, their clinical application is substantially constrained by the occurrence of adverse effects, including respiratory depression and addiction (3). In addition to their roles in anesthesia and analgesia, opioids have additional pharmacological effects, with distinct advantages, particularly in safeguarding organs against ischemia/reperfusion (I/R) injury. A number of opioids have been identified as candidate agents for clinical cardioprotection, with remifentanil being a case in point (4).

Remifentanil (RF) is an ultrashort-acting pharmacokinetic opioid developed by Feldman and his research team in 1991. It was synthesized by attaching a side chain containing a methyl ester to the piperidine ring nitrogen atom of fentanyl, to address the limitations of traditional opioids such as fentanyl—specifically, their prolonged duration of action and propensity for accumulation (5). RF shares a similar chemical structure to fentanyl, alfentanil, and sufentanil, and possesses analgesic potency comparable to fentanyl, being 100 times more potent than morphine (6, 7). As an opioid, RF exerts its pharmacological effects via the activation of OPRs. At clinical doses, RF has high affinity for μ-opioid receptors; when RF is administered at doses exceeding the clinical range, it can also interact with κ- and δ-opioid receptors (8). Unlike that of conventional opioids, the metabolic transformation of RF *in vivo* is independent of hepatic function. Instead, it undergoes rapid metabolism by nonspecific esterases present in the bloodstream and tissues, endowing it with the pharmacokinetic characteristics of rapid onset of action and a short half-life (9). Owing to these characteristics, RF is extensively employed in clinical practice for the induction and maintenance of anesthesia. With the discovery of the cardioprotective effects of opioids, many studies have reported the protective role of RF against organ ischemia–reperfusion injury, such as myocardial ischemia–reperfusion injury and cerebral ischemia–reperfusion injury. The mechanisms underlying its protective effects are associated with the attenuation of inflammation, the activation of antiapoptotic signaling pathways, and the inhibition of oxidative stress (4). In recent years, RF not only demonstrates distinct advantages in cardiovascular surgeries but also can be applied in anesthesia for various noncardiac surgical procedures, such as neurosurgical and spinal surgeries. Furthermore, it can be safely administered to patients across different age groups, including neonates, infants, and elderly individuals (10, 11).

In recent years, the neuroprotective effects of RF within the CNS have attracted the attention of researchers. This article systematically reviews the neuroprotective effects and underlying mechanisms of RF in acute brain injury and peripheral nerve-related disorders while further exploring the existing evidence and future challenges regarding its translation from basic research to clinical practice.

## 2 Literature search strategy

To comprehensively evaluate the neuroprotective effects of remifentanil, we conducted a systematic literature search. All literature published up to September 15, 2025, was retrieved from PubMed, Embase, Web of Science, and Google Scholar. The search query was: ((Neuroprotective Effects) OR (Neuroprotection) OR (brain protection)) AND ((Remifentanil) OR (Remifentanil hydrochloride) OR (Ultiva)). The initial database search yielded 86 records. After excluding review articles and duplicate records, 49 records were selected based on title and abstract screening. Following full-text reviewing of the most relevant literature, 28 studies were ultimately included for in-depth analysis and synthesis in this narrative review. Clinical study inclusion criteria: Randomized controlled trials conducted in patients with neurological disorders (e.g., traumatic brain injury) receiving remifentanil sedation/analgesia or at risk for neurological complications were included. Primary outcome measures comprised neurological function scores, visual analog scores, and postoperative delirium incidence. Basic research inclusion criteria: Mechanistic studies investigating remifentanil's neuroprotective or neurotoxic effects in experimental models of neurological disorders (e.g., animal or cellular models of cerebral ischemia-reperfusion injury, hypoxic-ischemic encephalopathy, neuroinflammation) were included.

## 3 Neuroprotective effects of remifentanil in basic research

### 3.1 Cerebral ischemia

Ischemic stroke refers to a neurological deficit syndrome caused by local cerebral circulatory disorders. As one of the major diseases endangering human health, cerebral ischemia can induce cerebral infarction, brain tissue necrosis, and focal neuronal damage (12). Significant neuronal injury occurs not only in the ischemic state but also after treatment with thrombolysis or mechanical thrombectomy. Therefore, the protection and regeneration of neurons have long been the focus of research on cerebral ischemia-related diseases. Studies have demonstrated that the neurological damage induced by cerebral ischemia is directly associated with neuronal loss, oxidative stress, and immune responses (12). Consequently, neurological damage caused by cerebral ischemia can be ameliorated through multiple approaches, such as enhancing neuronal protection, promoting neuronal repair and regeneration, directly mediating signaling pathways involved in neuronal survival and apoptosis, and regulating immune responses. In recent years, RF, an anesthetic and analgesic agent, has been shown to exert a protective effect against neuronal injury induced by cerebral ischemia–reperfusion (4) (Table 1).

**Table 1:** The neuroprotective effects of RF in experimental studies.

**Disorders**	**Animals/cells**	**Treatments (RE)**	**Functions**	**Mechamisms**	**Ref**.
Cerebral ischemia	Male SD rats	0.6 μg/kg/min for 5 min *i.v*.	Improved the spatial learning and memory; attenuated neuronal apoptosis in hippocampus	Activation of PI3K signaling pathway	(13)
	Male SD rats	5 μg/kg/min for 100 min, femoral vein	Improved the functional outcome and reduced the infarct volumes	Suppressed the phosphorylation of ERK 1/2 and TNF-α	(14)
	Adult male SD rats	1.2 μg/kg/min *i.v*.	Improve brain function and decrease the brain damage	Inhibit TNF-α/TNFR1, JNK signal transduction pathways	(15)
	SD rats	2 or 10 μg/kg, caudal veins	Decrease neuronal apoptosis level and cerebral infarct size, and increase the mitochondrial membrane potential	Repress the NR2B/CaMKIIα signaling pathway	(16)
	Male mongolian gerbils (11–13 weeks)	0.02 mg/kg *i.p*.	Alleviated ischemia-induced memory impairment	The suppression of apoptotic neuronal cell death	(17)
Neuroinflammation	Female Wistar Albino rats	0.04 mg/kg for 40 min *i.v*.	Prevent neuroinflammation, and apoptosis	Regulating the PI3K/AKT/HIF-1α pathway;restoring blood–brain barrier	(27)
	Male DDY mice; rat glioma cell line C6	10 μg/kg/min for mice; 100, 10, 1 μg/ml for cell	Suppressed increase in IL-6 mRNA levels in the brain in an inflammatory state	Inhibiting cyclic AMP synthesis	(29)
	BV2 microglia	0.5, 1, 2 μM	Suppressed PC12 cell injury	Suppressed inflammatory releases, iNOS, NO and PGE2 stimulated	(31)
Hypoxie-ischemic encephalopathy	HIBD rat model (SD, female, 6 weeks old)	5 mg/min/kg for 5 min	Improved the learning memory ability, reduced neuronal cell damage and apoptosis, reduced inflammation	Suppressing the expression of BTB domain and CNC homolog 1 (BACH1)	(36)
	Postnatal day 2 (P2) mice	500 ng/g for 10-min *i.p*.	Reduce excitotoxicity, the size of ibotenate-induced brain lesion; prevention of some behavioral deficits	Decreased ROS production, cortical caspase activity, DNA fragmentation, interleukin-1β levels, and reactive astrogliosis.	(37)
	Postnatal day 7 SD rat	5, 20, 80 μg/kg/h for 4 h	Reduces isoflurane-induced apoptosis	N.A.	(38)
	Cerebral slices from postnatal day 2 mice	50 μM	Inhibited apoptosis and preserved mitochondrial integrity	Activate the opioid and NMDA receptors, and the mitochondrial-dependent apoptotic pathway	(39)
	Olfactory neurons from newborn SD rat	2-, 0.2- or 0.02-mM	Reduce glutamate toxicity and to increase cell viability	N.A.	(40)
Surgery-related brain injuryury	94 healthy adult male SD rats	1.2 μg/kg/min	Attenuated CPB-induced injury	Blocking AKT/NRF2 signal pathway	(44)

With in-depth investigations into the neuroprotective effects of RF in cerebral ischemia, its underlying mechanisms have gradually become the focus of exploration. A study conducted by Hu et al. (13) demonstrated that RF administration could enhance spatial learning and memory abilities in rats after cerebral ischemia while attenuating hippocampal neuronal apoptosis. Further experiments revealed that the application of a PI3K inhibitor abrogated both the RF-mediated improvement in neurological function and the antiapoptotic properties of RF in postischemic rats, suggesting that the neuroprotective effect of RF against cerebral ischemia–reperfusion injury may be exerted through the activation of the PI3K pathway. Similarly, Jeong et al. (14) reported that RF could reduce cerebral infarct volume and decrease neurological deficit scores in rats following cerebral ischemia. Mechanistic studies indicated that RF significantly downregulated the expression of phosphorylated ERK 1/2 and TNF-α while increasing the expression of δ-opioid receptors in the rat brain. Zhang et al. (15) reported that RF treatment alleviated cerebral ischemia-induced brain damage in rats; in the RF-treated group, the protein expression levels of TNF-α, TNFR1, Cytc, caspase-3, caspase-9, and pJNK in rat brain tissues were downregulated. These findings suggest that RF may exert its neuroprotective effects by inhibiting the mitochondrial apoptotic pathway (TNF-α/TNFR1) and the JNK signaling pathway. By establishing a cerebral I/R rat model, Chen et al. (16) discovered that RF could reduce neuronal apoptosis and cerebral infarct size in rats, along with decreasing ROS levels and the MDA content. Further studies revealed that RF treatment significantly reduced the expression levels of cell death-related proteins (e.g., caspase-3) and proteins associated with the NR2B/CaMKIIα signaling pathway in rat brain tissues, indicating that RF can exert neuroprotective effects by inhibiting the NR2B/CaMKIIα signaling pathway and suppressing neuronal apoptosis in rats with cerebral ischemia–reperfusion injury (IRI). Research by Park et al. (17) revealed that RF could ameliorate cognitive impairment in adult male gerbils after transient cerebral ischemia. Memory function was evaluated via the step-down avoidance task, and the results revealed that RF treatment significantly shortened the latency period in gerbils, which was associated with RF-mediated inhibition of hippocampal apoptosis.

In addition to exerting protective effects against cerebral ischemia, RF also exerts protective effects against IRI in multiple other organs, such as the myocardium (18), liver (19), and intestine (20). Notably, RF can ameliorate the neurological dysfunction associated with ischemic injury in these noncerebral organs. One study revealed that the hepatoprotective effect of RF in rats with hepatic IRI is associated with the activation of the dorsal motor nucleus of the vagus nerve (DMV), rather than peripheral μ-opioid receptors, suggesting an interaction between RF and the central parasympathetic nervous system (21). Another study reported that RF protects against hepatic ischemia/reperfusion-induced injury to D1 medium spiny neurons (D1-MSNs) (22). Specifically, this protective effect manifests as downregulated expression of IL-1β and IL-18, attenuated oxidative stress, improved morphology and function of D1-MSNs, and reduced apoptosis. This protective mechanism is believed to be associated with RF-mediated upregulation of FGF18 expression, and FGF18 may serve as a potential therapeutic target for hepatic I/R-related neurological injury.

**Figure 1:**
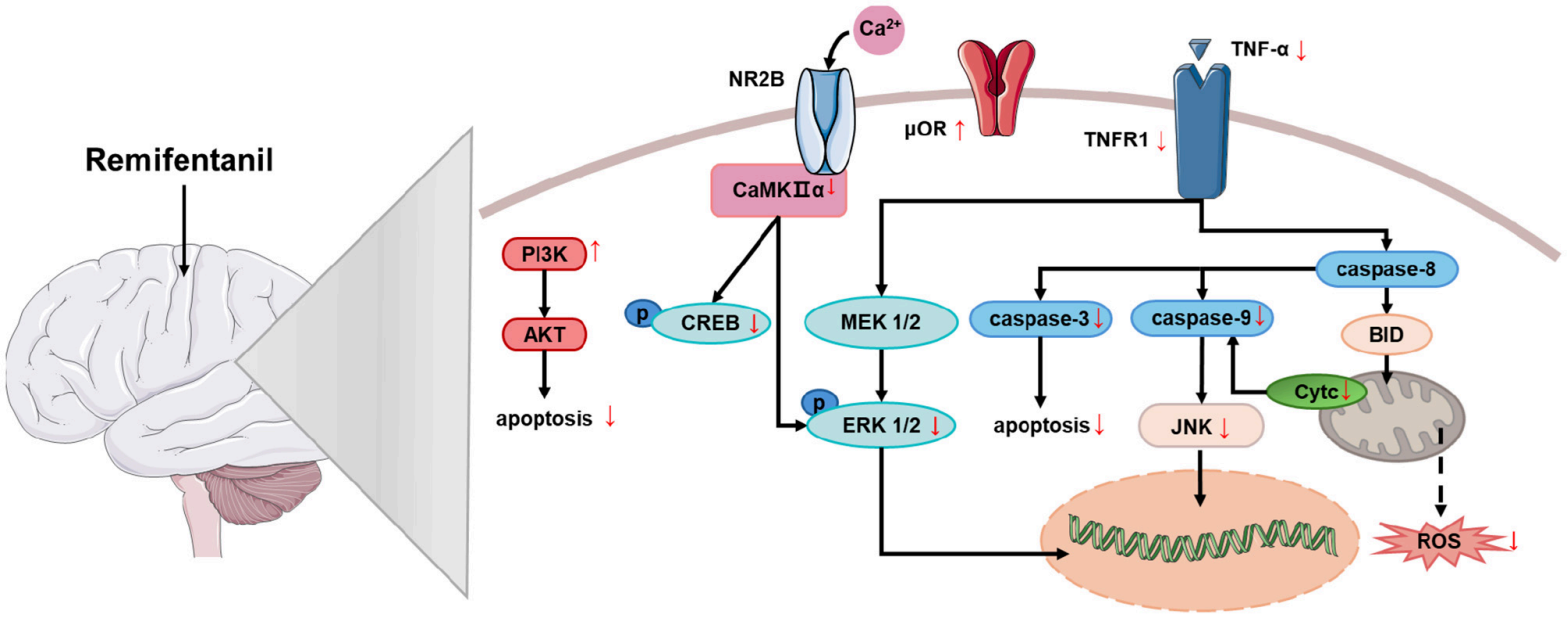
The mechanism of the neuroprotective effect of RF on cerebral ischemia. Remifentanil exerts neuroprotective effects against cerebral ischemia by mediating multiple signaling pathways that reduce apoptosis and oxidative stress. PI3K, phosphoinositide 3-kinase; Akt (PKB), protein kinase B; NR2B (GluN2B), NMDA receptor subtype 2B; CaMKIIα, calcium-dependent protein kinase II alpha; CREB, cAMP response element-binding protein; MEK 1/2 (MAP2K), mitogen-activated protein kinase kinase 1/2; ERK 1/2, extracellular signal-regulated kinase 1/2; μOR, mu-opioid receptor; TNFR1, tumor necrosis factor receptor 1; TNF-α, tumor necrosis factor-alpha; Caspase-3/8/9, cysteine-dependent aspartate-specific protease-3/8/9; JNK, c-Jun N-terminal kinase; Cyt c, cytochrome c; Bid, BH3-interacting domain death agonist; ROS, reactive oxygen species.

The aforementioned studies demonstrate that RF exerts a certain degree of protection against cerebral injury induced by cerebral ischemia/reperfusion (Table 1), and existing research has revealed that RF exerts its effects through multiple signaling pathways (Figure 1). Additionally, RF can ameliorate cognitive impairment induced by cerebral ischemia and protect against neurological injury associated with ischemia–reperfusion in other organs (4, 17).

### 3.2 Neuroinflammation

Neuroinflammation constitutes a crucial pathological process in a spectrum of CNS disorders, such as Alzheimer's disease and Parkinson's disease (23). This process involves a cascade of complex responses, including the activation of neuroglial cells, the release of inflammatory mediators, and the generation of ROS (24). Under physiological conditions, neuroinflammation serves to protect the brain against pathogenic insults. However, sustained neuroinflammation also leads to neuronal damage (25). Notably, anesthetics and sedatives are recognized to affect cerebral inflammation. Opioids, ketamine, benzodiazepines, and propofol can elicit both neuroprotective and neurotoxic effects in the brain, with such outcomes being dependent on the duration of administration and dosage employed (26).

RF can ameliorate LPS-induced neuroinflammation (27). In the LPS-induced sepsis rat model, cerebral edema and inflammatory cell infiltration were observed, and the expression levels of PI3K, AKT, and HIF-1α were significantly elevated. However, all these pathological manifestations and molecular changes were markedly attenuated in the RF-treated group. As a crucial downstream substrate of the PI3K/AKT pathway, HIF-1α plays a pivotal role in regulating blood–brain barrier (BBB) permeability (28). These findings indicate that RF has a therapeutic effect on alleviating sepsis-associated neuroinflammation, and its underlying mechanism is associated with the downregulation of the PI3K/AKT/HIF-1α pathway and the restoration of BBB integrity. A study conducted by Maeda et al. (29) directly measured the expression level of the proinflammatory cytokine IL-6 in mice following LPS induction. The results demonstrated that RF significantly reduced the IL-6 mRNA levels in the hypothalamus, cortex, and plasma of the mice. *In vitro* cellular experiments revealed that RF inhibited IL-6 expression by suppressing the intracellular increase in cAMP in neuroglial cells. However, the mechanistic investigation has been insufficient, as the study failed to establish a direct causal link between RF-mediated cAMP inhibition and the reduction in IL-6 mRNA expression. Previous research has reported that the cAMP/PKA pathway mediates IL-1β-induced IL-6 synthesis, which is associated with the upregulation of the JAK2/STAT3 pathway (30). Therefore, subsequent studies could explore the role of the JAK2/STAT3 pathway in neuroglial cells following RF treatment to further clarify this mechanism. Another study similarly indicated that RF can inhibit the release of inflammatory factors from LPS-induced BV2 microglia (31). The findings of this study revealed that RF suppressed the LPS-induced increase in TNF-α, IL-6, iNOS, NO, and PGE2 in BV2 cells, and the inhibitory effect was positively correlated with RF concentration. Additionally, RF treatment led to significant reductions in the protein levels of p-NF-κB p65, p-IκBα, and COX2 in the cells. These results suggest that RF can reduce the release of inflammatory factors by inhibiting the NF-κB signaling pathway. Collectively, the aforementioned studies illustrate that RF mitigates LPS-induced neuroinflammation. Its protective mechanisms involve the downregulation of the PI3K/AKT/HIF-1α pathway and NF-κB signaling, the restoration of BBB integrity, and the suppression of intracellular cAMP expression in neuroglial cells (Table 1).

### 3.3 Neonatal hypoxic-ischemic encephalopathy (NHIE)

Neonatal encephalopathy (NE) refers to a spectrum of acute neurological dysfunctions characterized by altered states of consciousness, abnormal reflexes, convulsions, and other manifestations and occurs in neonates with a gestational age of ≥35 weeks (32). Hypoxia-ischemia is the most common etiological factor of NE; in addition, other factors, such as traumatic brain injury, bacterial or viral infections, and vascular disorders, may also induce NE (33, 34). Perinatal/neonatal hypoxic-ischemic encephalopathy (HIE) represents one of the most severe conditions encountered in neonatal neurology and is also among the leading causes of neonatal mortality and morbidity (35). Lu and Wang study (36) demonstrated that RF exerts neuroprotective effects by establishing a rat hypoxic-ischemic brain damage (HIBD) model. These effects specifically manifested as reductions in neuronal damage and apoptosis, decreases in inflammation, and improvements in the learning and memory abilities of HIBD rats. Further mechanistic investigations revealed that RF can downregulate the expression of BTB and CNC homology 1 (BACH1), thereby inhibiting the expression of TNF receptor-associated factor 3 (TRAF3). KEGG enrichment analysis revealed that TRAF3-related genes are enriched in the NF-κB signaling pathway. These findings suggest that RF can alleviate HIBD-induced cognitive impairment by downregulating BACH1 expression to inhibit the TRAF3/NF-κB signaling pathway. In another study, an ibotenate-induced neonatal brain injury model was established, and RF also reduced the production of ROS, the level of IL-1β, and the number of reactive astrocytes in the injured neonatal brain while enhancing relevant motor abilities, thus demonstrating a protective effect against neonatal brain injury (37). In a study conducted by Pan et al. (38), 7-day-old rats were subjected to continuous isoflurane exposure. The results showed that RF administration alone did not induce cell death; instead, it attenuated isoflurane-induced hippocampal apoptosis in developing rats. Tourrel et al. (39) investigated the effects of RF on the immature mouse brain. Brain slices isolated from postnatal day 2 (P2) mice were exposed to RF, and the results demonstrated that RF at a concentration of 50 μM had no necrotic effects but did have antiapoptotic effects *in vitro*. This antiapoptotic effect was abrogated by opioid receptor antagonists and NMDA receptor antagonists, suggesting that the underlying mechanism involves opioid ligands, NMDA receptors, and the mitochondria-dependent apoptotic pathway. Additionally, RF was found to attenuate glutamate-induced neuronal damage in the olfactory bulb of neonatal rats and increase neuronal cell viability (40). Collectively, these studies indicate that RF confers a certain degree of protection against neonatal-related encephalopathies (Table 1), with mechanisms involving the inhibition of BTB and CNC homology 1 (BACH1) expression, downregulation of the TRAF3/NF-κB signaling pathway, suppression of apoptosis, mitigation of oxidative stress, and alleviation of inflammation.

### 3.4 Surgery-related brain injury

Surgical intervention constitutes a prevalent therapeutic modality in the advancement of modern medicine, and surgical anesthesia is also recognized as a risk factor for cerebral injury. Patients who undergo surgery are susceptible to adverse outcomes such as stroke, postoperative delirium, and postoperative cognitive dysfunction, which are associated with factors such as surgical type and patient physical status. Research findings have revealed that the incidence of cerebral injury in patients undergoing cardiac surgery is significantly greater than that in those undergoing other noncardiac surgical procedures (41). Up to 40% of patients who undergo cardiopulmonary bypass (CPB) surgery develop perioperative neurological injury, which severely impairs postoperative recovery (42). A study conducted by Xiong et al. (43) demonstrated that the preoperative administration of RF to rats subjected to CPB mitigated surgery-induced oxidative stress, inflammation, and cerebral injury; shortened the latency of escape responses; improved memory function; and reduced hippocampal neuronal apoptosis in these rats. Further mechanistic investigations indicated that preoperative RF treatment reversed the CPB-induced downregulation of p-Akt and HO-1 expression and further inhibited the expression of Nrf2. These results suggest that the ameliorative effect of RF on CPB-induced cerebral injury in rats is associated with the Akt/Nrf2 signaling pathway. Studies have demonstrated that the PI3K/Akt/Nrf2/HO-1 signaling pathway plays a crucial role in cerebral injury associated with various neurological disorders, such as epilepsy and Alzheimer's disease (44, 45). The activation of this pathway is closely linked to pathological processes, including oxidative stress, inflammatory responses, and apoptosis (46). Currently, there are few research reports regarding the effects of RF on cerebral injury induced by other types of surgeries. Therefore, further basic research focusing on the neuroprotective mechanisms of RF in this field is warranted in future studies.

## 4 Neuroprotective effects of RF in clinical practice

### 4.1 Traumatic brain injury (TBI)

TBI refers to substantial damage to brain tissue, which can impair brain function either transiently or persistently. TBI often leads to severe neurological impairment and psychological distress, such as cognitive decline (47). The primary focus of initial TBI management is maintaining a patent airway, along with sustaining adequate ventilation, oxygenation, and blood pressure support. For patients with severe TBI, surgical intervention is frequently needed, followed by intensive postoperative monitoring and intracranial pressure surveillance. In recent years, therapeutic strategies involving pharmacotherapy for TBI have emerged as a research focus, aiming to enhance cognitive and neurological function recovery through neuroprotection and neural restoration (48). Endogenous opioids are considered to play a role in the pathophysiology of TBI, and the administration of opioids can influence brain injury and provide neuroprotection following TBI (49). A study conducted by Karabinis et al. (50) revealed that in patients with acute brain injury or those intubated post-neurosurgery, the administration of RF significantly shortened the time required for neurological assessment (*P* < 0.001 vs. fentanyl and morphine), and the extubation rate was notably faster than that in the morphine-treated group. These findings indicate that RF has superior analgesia-based sedative effects and enables more rapid awakening for neurological evaluation. Another study similarly demonstrated that in pediatric patients hospitalized due to TBI, continuous infusion of RF (at a median dose of 0.25 μg/kg/min) achieved target levels of sedation scores and neurological status, suggesting that RF can provide sedation with rapid onset and facilitate prompt recovery (51). A study by Engelhard et al. (52) also indicated that when used as an analgesic in TBI patients, RF did not exert adverse effects on cerebrovascular hemodynamics, cerebral perfusion pressure, or intracranial pressure. Collectively, these findings suggest that continuous RF infusion constitutes a safe and effective analgesic regimen for TBI patients or other patients intubated postneurosurgery. Moreover, RF has significant advantages in neurological assessment within clinical settings. In the future, developing an RF-centered analgesic regimen for TBI is promising, thereby enabling earlier intervention and improving the long-term neurological prognosis of patients (Table 2).

**Table 2:** The neuroprotective effects of RF in clinical.

**Disorders**	**Ages and genders**	**Control group**	**Remifentanil group**	**Clinical outcome**	**Ref**.
TBI	Aged 18–80 years, *n* = 161	Fentanyl (*n* = 37) or morphine (*n* = 40)	*n* = 84, before the propofol infusion started at 9 μg/kg/h	Permitted significantly faster and more predictable awakening for neurological assessment.	(50)
	Median age was 9 years, *n* = 38	Self-control	0.25 μg/kg/min	Neurologic examinations remained reassuring during remifentanil infusion, a suitable sedative agent for use in children with TBI	(51)
	Aged 46 ± 18 years, *n* = 20	Self-control	0.5 mg/kg followed by 0.25 mg/kg/min for 20 min	No change in mean arterial blood pressure, intracranial pressure, and cerebral blood flow velocity	(52)
Delirium	752 patients >18	Fentanyl (*n* = 376)	Intraoperative anesthetic dosage	Lower incidence of post-operative delirium in the early post-operative period	(57)
	80 children aged 3–7 years	Intraoperative remifentanil infusion (0.2 μg/kg/min)	Maintenance during the recovery phase 0.05 μg/kg/min	Attenuated the incidence of emergence delirium	(58)
	95 nonoperative patients requiring ventilatory opioids >18	Fentanyl, noradrenaline, midazolam, propofol, and dexmedetomidine	0.029 μg/kg/min	Reduce delirium duration	(59)
	84 patients aged 3–11 years	Sevoflurane and DEX (1 mg/kg)	Propofol (4 mg/kg/h) and remifentanil (0.03 mg/kg/h)	Recovery time was significantly shorter; incidence of emergence delirium decreased in both groups	(60)
Spinal cord injury	60 patients aged 14–28	Alfentanil (1 mg/kg/min)	0.2 mg/kg/min	Wake-up test can be conducted faster with remifentanil during PSF surgery.	(66)
Surgical-related brain injury and tracheal intubation	Aged 55–65 years, *n* = 40	Saline	0.6, 1.2, and 1.8 μg/kg/min	Played a protective role on brain damage, inhibition of the oxidative stress response	(70)
	2,760 patients underwent clipping of ruptured/unruptured ICA	Fentanyl only	Fentanyl+remifentanyl; anesthetic dosage	Reduced mortality	(71)
	160 patients >18	Saline	0.5 and 0.05 μg/kg/min for 20 min	Significantly reduces the incidence of severe pain; the visual analog score is significantly lower.	(73)
	161 patients aged 18–80 years	Fentanyl (*n* = 37) or morphine (*n* = 40)	9–18 μg/kg/h	Significantly faster and more predictable awakening for neurological assessment	(50)
	91 patients >18	Fentanyl	1 mg/kg and 1 mg/kg/min	Time to preoperative neurological recovery is faster	(74)
Postoperative cognitive dysfunction	70 patients undergoing radical surgery for cervical cancer	Fentanyl (*n* = 35) 0.3 μg/kg/min; maintenance dose of 1 μg/kg	0.3 μg/kg/min; maintenance dose of 3 μg/kg	Low occurrence of postoperative cognitive dysfunction	(76)

### 4.2 Delirium

Delirium is an acute brain dysfunction characterized by impairments in cognitive ability, attention, and memory function, accompanied by symptoms such as disturbance of consciousness, disorientation, sensory confusion, and agitation. This condition is highly prevalent among hospitalized elderly patients, with an incidence of up to 50% following high-risk surgical procedures and as high as 75% in critically ill patients requiring mechanical ventilation (53). Currently, there is no single intervention or pharmacotherapeutic agent for the treatment of delirium; clinical management primarily involves addressing the underlying cause, controlling psychiatric symptoms, and promoting cognitive-enhancing factors (54). Opioids, as commonly used clinical analgesics, are a major contributing factor to the development of postanaesthetic delirium (55). A study investigating the impact of different opioids on delirium incidence revealed that the use of tramadol or pethidine was associated with an increased risk of delirium, whereas no such association was observed with the use of morphine, fentanyl, oxycodone, or codeine (56). As a novel synthetic opioid, RF has a lower incidence of delirium than other opioids, such as fentanyl (Table 2). An analysis of 752 clinical cases demonstrated that the use of different intraoperative anesthetic opioids was associated with variations in postoperative delirium incidence, with the RF group showing a lower rate of early postoperative delirium than the fentanyl group (57). A study by Choi et al. (58) reported that, in children who underwent strabismus surgery under sevoflurane anesthesia, maintaining low-dose RF infusion during both the intraoperative period and recovery phase effectively reduced the incidence of postoperative delirium compared with intraoperative RF infusion alone. In addition to postoperative delirium following conventional surgery, another study revealed that RF also has a beneficial effect on delirium associated with mechanical ventilation in nonsurgical ICU patients. Compared with non-RF anesthetics such as fentanyl, RF significantly shortens the duration of delirium and effectively alleviates agitation (59). A study by Oriby and Elrashidy (60) compared total intravenous anesthesia with propofol and RF vs. inhalational sevoflurane combined with dexmedetomidine in children undergoing strabismus surgery with respect to emergence delirium. The results revealed no difference in the incidence of emergence delirium between the two groups; however, the propofol-RF group presented a significantly shorter recovery time. Collectively, these studies indicate that, compared with other sedatives, including fentanyl, RF reduces the incidence of delirium and shortens its duration in patients. Furthermore, maintaining RF infusion during the postoperative recovery phase results in a lower incidence of delirium than does intraoperative infusion alone (Table 2). These findings have important implications for the clinical selection of analgesic agents and the development of administration strategies for patients at high risk of delirium.

### 4.3 Spinal cord injury

Spinal cord injury (SCI) is a severe CNS disorder caused by external physical force, impairing patients' sensory and motor functions while inducing multisystem complications (e.g., psychological disorders) with poor prognosis (61). Secondary SCI results from persistent spinal cord compression, involving damage to neural tissue or vascular structures, followed by hypoxia and localized ischemic infarction after blood supply disruption, exacerbating spinal cord tissue damage (62). Secondary injury is a complex process involving mechanisms such as inflammatory response, oxidative stress, and neuronal apoptosis (63). Current clinical management of SCI primarily focuses on acute decompression surgery and postoperative rehabilitation training, with limited neuroprotective interventions targeting neuropathic pain.

Experimental studies have demonstrated that RF induces hyperalgesia or pro-nociceptive effects in mice or rat by changing the expressions of genes or proteins in the spinal cord (64, 65). For example, RF exerted pro-nociceptive effects in mice, which were similar to surgical injury. DOR mRNA levels in the dorsal root ganglia were inhibited by either RF administration or surgery. Downregulating DOR contributes to RF and surgery-induced nociception, while enhancing enkephalin levels in the spinal cord and the periphery mostly reversed postoperative pain that is caused by RF (64). In a rat model with plantar incision surgery, the continuous infusion of RF resulted in elevated P2X4 and BDNF expression, which played essential roles in RF-induced hyperalgesia (65).

A randomized controlled trial involving 60 patients undergoing elective posterior thoracolumbar spinal fusion (PSF) found that intraoperative RF infusion significantly shortened patient recovery time compared to alfentanil (66) (Table 2). Although this study indicated potential benefits of RF for SCI, it compared only recovery time and quality of recovery without assessing commonly used neurological methods for detecting intraoperative spinal cord dysfunctions, e.g., somatosensory evoked potentials and motor evoked potentials, representing a limitation. A prospective, randomized, controlled clinical trial indicated that intraoperative anesthesia with dexmedetomidine and lidocaine in patients undergoing multi-segmental spinal fusion significantly reduced postoperative nausea and vomiting incidence and decreased morphine consumption within 24 h postoperatively. The fentanyl treatment and RF group further reduced the probability of requiring antihypertensive medications and shortened hospital stay (67). Another double-blind study indicated that intraoperative use of RF combined with low-dose ketamine during spinal fusion surgery improved hemodynamic stability and reduced postoperative morphine consumption (compared to RF infusion alone) (68). These studies demonstrate that RF, as a primary anesthetic agent in spinal surgery, provides superior hemodynamic stability, reduces the risk of secondary injury, significantly decreases postoperative opioid consumption, and delivers effective analgesia.

### 4.4 Surgery-related cerebral injury and tracheal intubation

Surgical cerebral injury refers to the unavoidable brain damage induced by various surgical manipulations during neurosurgical procedures, with specific contributing factors, including incisions, retraction, electrocautery-induced thermal injury, and intraoperative hemorrhage (69). In addition to these neurosurgical interventions, studies have indicated that cerebral injury is relatively common in patients undergoing cardiac surgery (41). A study by Zhang et al. (70) demonstrated that preoperative administration of RF in patients undergoing pump-assisted coronary artery bypass grafting mitigated oxidative stress responses, significantly reduced S-100β protein levels, and exerted a protective effect against cerebral injury. This finding is consistent with the aforementioned role of RF in attenuating CPB-induced cerebral injury in rats. A retrospective study compared postoperative complications, in-hospital mortality, and length of hospital stay among 1,380 pairs of patients who underwent intracranial aneurysm clipping. The results revealed that the in-hospital mortality rate in the RF + fentanyl group was significantly lower than that in the fentanyl-only group; although there was no significant difference in the incidence of postoperative complications between the two groups, the severity of hydrocephalus was alleviated in the RF + fentanyl group (71). This study revealed that RF administration is an independent factor contributing to the reduction in postoperative in-hospital mortality in ICA patients, which provides certain implications for the clinical selection of opioid analgesics in this type of surgery.

Neuromonitored tracheal intubation is a medical technique that integrates airway management with neurological function monitoring. It is utilized primarily in surgical procedures or intensive care settings to enable real-time assessment of patients' neurological status while ensuring airway patency and effective ventilation (72). Neurosurgical patients often experience significant pain and stress responses during tracheal intubation; thus, analgesic and sedative agents are commonly employed in this scenario. A study revealed that when neurosurgical patients received either RF or normal saline before extubation, the incidence of severe pain in the RF group during extubation was significantly reduced, with no statistically significant difference in immediate vital signs between the two groups. These findings indicate that prophylactic RF administration can safely and effectively reduce the incidence of pain associated with tracheal intubation in neurosurgical patients (73). Another study compared the effects of RF, fentanyl, and morphine on cerebral injury in patients with acute brain injury or those intubated after neurosurgery. The results showed that with RF use, the variability in patients' neurological assessment results (before and after evaluation) was significantly reduced, the average time required for neurological assessment was notably shorter, and the extubation speed was significantly faster than that in the morphine group (50). A study by Gelb et al. (74) demonstrated that RF serves as a safe alternative to fentanyl in supratentorial craniotomy. RF administration led to a reduction in patients' systolic blood pressure; although there was no difference in extubation time between the two groups, patients in the RF group achieved faster preoperative mental recovery. Collectively, these findings suggest that RF provides effective and safe analgesic effects for neurosurgical patients undergoing surgery and tracheal intubation. Compared with fentanyl, RF has potential advantages in alleviating neurological injury in patients (Table 2).

### 4.5 Postoperative cognitive disorders

Postoperative cognitive dysfunction (POCD) represents a prevalent CNS complication subsequent to surgery and anesthesia, with its core clinical manifestations encompassing memory impairment, attentional deficits, disorientation, reduced executive function, and diminished language fluency. Such cognitive impairments not only impede patients' postoperative recovery trajectory and prolong hospital stay but are also are closely associated with an elevated risk of postoperative mortality (75). A research team led by Lu et al. (76) compared the effects of intraoperative intravenous infusion of RF vs. fentanyl on postoperative cognitive function and inflammatory responses in patients undergoing radical hysterectomy for cervical cancer. The findings revealed that the postoperative awakening time in the RF group was significantly shorter than that in the fentanyl group, and the incidence of POCD in the RF group was notably lower than that in the fentanyl group (Table 2). Despite the aforementioned positive effects of RF on cognitive dysfunction, several studies have also documented its adverse impacts on cognitive function. One study reported that the administration of propofol, RF, or their combination in SD rats induced cognitive decline, which was specifically characterized by a significant prolongation of the latency period, as assessed by the Morris water maze test, and an increased level of Tau protein phosphorylation in the rat hippocampus (77). Another prospective, double-blind, randomized study reported no significant difference between RF and fentanyl in the incidence of POCD in elderly patients who underwent major abdominal surgery (78). Although the level of IL-6 in the RF group was significantly lower on the 7th postoperative day and the incidence of POCD in this group was the lowest among all patients, the absence of a direct correlation between inflammatory factor levels and POCD, coupled with the nonsignificant difference in POCD incidence, precluded the confirmation that RF reduces POCD in patients undergoing abdominal surgery. Studies focusing on opioids have demonstrated that morphine, fentanyl, and other opioids play pivotal roles in the inflammatory response (79). Notably, the aforementioned study (involving major abdominal surgery) indicated that RF has superior anti-inflammatory efficacy to fentanyl. This particular study enrolled 622 patients aged over 60 years who underwent major abdominal surgery as experimental subjects, indicating that the scale and research value of the scale are certain. In contrast, the study by Lu et al. (76) included 70 female patients who underwent radical hysterectomy for cervical cancer. The conflicting conclusions drawn from the two studies may be attributed to inconsistencies in the definition and assessment tools for POCD, as well as differences in the timing of evaluations. Furthermore, variations in clinical sample size, surgical procedures, patient gender, and age may have limited the statistical power and generalizability of the results, potentially introducing selection bias. Both studies compared the effects of RF and fentanyl on the incidence of POCD without conducting an in-depth exploration of other potential mechanisms or a broader range of neurocognitive indicators. Nevertheless, these studies still provide valuable references for the selection of intraoperative anesthetic and analgesic agents aimed at preventing POCD in different types of surgical procedures.

## 5 Remifentanil-induced hyperalgesia

Opioids are commonly used to treat neuropathic pain. Woller and Hook (80) found that clinical opioid use may increase pain progression, reduce motor recovery, and elevate infection risk. Opioids, including morphine (81), fentanyl (82), and heroin (83), can induce opioid-induced hyperalgesia (OIH), an adverse reaction that cannot be overcome by increasing drug dosage. In-depth investigation of OIH mechanisms and enhanced clinical management of opioid use are key research priorities.

Remifentanil-induced hyperalgesia (RIH) represents one of the most common adverse reactions associated with the clinical use of RF, and the mechanisms underlying RF-induced hyperalgesia have emerged as a research focus in recent years (84). Generally, the occurrence of RIH is reportedly associated with continuous RF infusion (at a rate of 0.3 mg/kg/min or higher) or abrupt discontinuation of RF (85). The mechanism of RIH involves sensitization of the central nervous system and is closely related to the activation of N-methyl-D-aspartate (NMDA) receptors (86). For example, the high-dose intravenous RF administration (bolus of 6.0 and 2.5 μg/kg/min for 2 h) induces hyperalgesia in rats, accompanied by elevated levels of phosphorylated CaMKII in the central nervous system, reflecting the interaction between NMDA receptors and pain mechanisms (87).

The core strategies for preventing and alleviating clinical RIH involve NMDA receptor antagonism and multimodal analgesia. A randomized controlled trial indicated that high-dose RF infusion (0.40 μg/kg/min) during major abdominal surgery led to postoperative hyperalgesia and increased morphine consumption, whereas low-dose ketamine (0.5 mg/kg) mitigated RIH by blocking NMDA receptors (88). A randomized controlled study involving 180 patients undergoing laparoscopic cholecystectomy found that propofol (0.3 μg/kg/min) suppressed NMDA glutamate subtype receptors and alleviated RIH by modulating NMDA receptor-mediated intracellular calcium influx (89). Beyond the NMDA pathway, a meta-analysis systematically evaluated preclinical studies on RIH mitigation, detailing comparative efficacy across different drug groups (RF dosage, animal models, etc.). Among these, Annexin A1-derived peptide (Anxa12-26), P2Y purinoceptor 1 antagonist (MRS2179), and α2-adrenergic agonist dexmedetomidine ranked highly and warrant inclusion in future clinical interventions for RIH (90).

RF has demonstrated to provide rapid recovery, reduce postoperative delirium, and mitigate secondary brain injury through hemodynamic stabilization in patients with TBI and surgery-related neurological damage. When considering RF for neuroprotection, its potential risk of RIH must be weighed. Thus, for clinical anesthesia and analgesia, RF administration requires careful dose selection, gradual tapering, and consideration of combination therapy (e.g., propofol, dexmedetomidine). For non-high-risk surgeries or chronic pain patients, dosage and duration must be cautiously evaluated.

## 6 Limitations

The main limitation of this review is related to the heterogeneity of clinical studies. This narrative review typically covers a broader range of literature concerning the neuroprotective effects of RF. However, each CNS disorder has its specific clinical manifestations, making it difficult to compare the neuroprotective effects of RF among different CNS diseases. The overall quality of those studies has not been evaluated qualitatively. Therefore, potential biases, such as literature selection bias, performance bias, detection bias, attrition bias, and reporting bias, cannot be completely avoided. To further confirm the neuroprotective effects of RN, more rigorously designed and large-scale prospective studies should be included for qualitative analysis and comparison.

Although RF has demonstrated significant neuroprotective effects in experimental models such as the rat MCAO model, these findings carry certain limitations. First, most preclinical studies utilize healthy animals, whose pathophysiological processes differ significantly from those of patients with multiple comorbidities (e.g., hypertension, diabetes), thereby limiting their clinical translation value. Future basic research must further investigate whether the neuroprotective signaling pathways of RF remain effective in complex models involving common clinical comorbidities such as advanced age, gender, metabolic disorders (e.g., hyperlipidemia and hyperglycemia), hypertension, and atherosclerosis.

## 7 Conclusion and perspective

**Figure 2:**
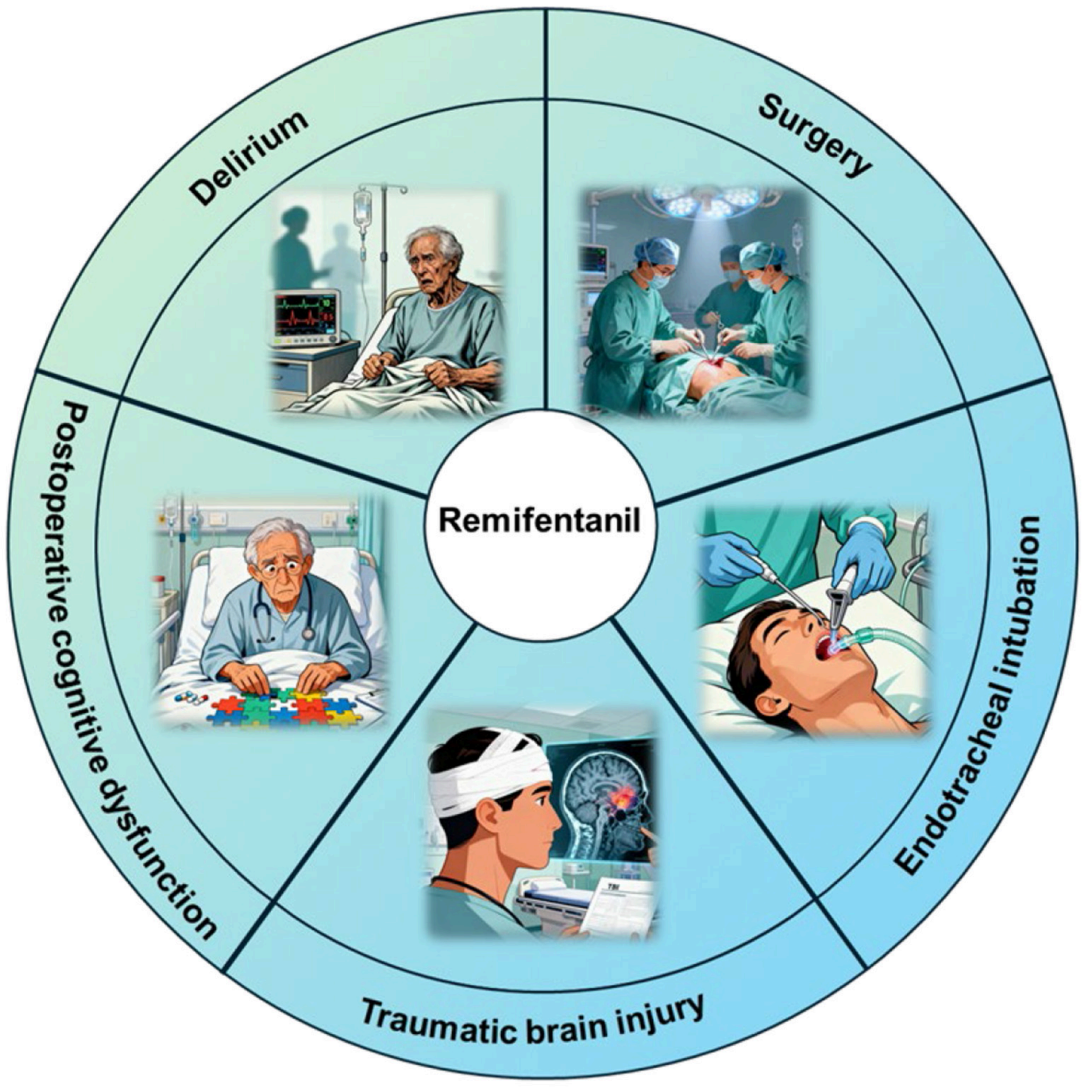
The neuroprotective effect of remifentanil in clinical applications. Remifentanil plays a neuroprotective role in traumatic brain injury, delirium, postoperative cognitive dysfunction, surgical procedures, and tracheal intubation.

As a synthetic ultrashort-acting opioid, RF has certain neuroprotective effects. Basic research has demonstrated that RF exerts protective effects against cerebral ischemia/reperfusion injury through mechanisms such as reducing inflammation, activating antiapoptotic signaling pathways, and inhibiting oxidative stress; RF also alleviates LPS-induced neuroinflammation both *in vivo* and *in vitro* and confers protective effects against neonatal encephalopathy and surgery-related cerebral injury. Clinical studies have shown that RF serves as a safe and effective analgesic regimen for patients with TBI, patients undergoing neurosurgical procedures, and patients requiring tracheal intubation while also providing certain benefits for the recovery of patients' neurological function (Figure 2). Additionally, compared with fentanyl, RF is associated with a reduced incidence of delirium and postoperative cognitive dysfunction in patients.

RF is most commonly used in surgical anesthesia. One study compared the use of RF, fentanyl, and alfentanil (each combined with propofol) for anesthetic induction and maintenance in patients undergoing craniotomy. The results revealed no significant differences among the three groups in terms of the required maintenance dose of propofol, heart rate, or mean arterial pressure. However, patients in the RF group presented a significantly shorter eye-opening time and achieved faster postoperative recovery (91). In conjunction with the aforementioned clinical findings, RF is associated with a lower incidence of postoperative delirium, surgery-related cerebral injury, and other adverse outcomes than fentanyl is. These differences are likely attributed to the significant pharmacokinetic disparities between RF and fentanyl. These studies suggest that in the clinical selection of anesthetic agents, RF may be considered one of the preferred analgesic regimens, depending on the type of surgery and the risk of neurological sequelae in patients. The clinical studies analyzed in this review demonstrate that RF offers clear advantages in specific clinical scenarios, such as intraoperative hemodynamic management and rapid recovery. However, adverse reactions with opioid medications, such as RIH, must be considered in clinical practice. Therefore, when RF is selected for clinical anesthetic analgesia, careful attention should be given to the injection dose, and considerations should be given to strategies such as gradual drug withdrawal and combined administration with other agents (e.g., propofol and dexmedetomidine). Future research should focus on in-depth investigations into the mechanisms of RF-induced hyperalgesia and the development of more effective and safe preventive approaches. Furthermore, it is essential to conduct in-depth studies on the relationship between RF dosage and its neuroprotective effects; explore the optimal timing (pretreatment, posttreatment, or full-course intervention), optimal dosage, and infusion mode for RF-mediated neuroprotection; and develop personalized medication regimens for RF in different populations (e.g., elderly individuals, neonates, and populations at high risk of neurodegenerative diseases).
